# Supplementary material for: Timescales of water accumulation in magmas and implications for short warning times of explosive eruptions
Source: Nat Commun. 2018 Feb 22;9:770. doi: 10.1038/s41467-018-02987-6 (PMC5823946; doi:10.1038/s41467-018-02987-6)
Supplement: Supplementary file 1 — Supplementary Information [file 41467_2018_2987_MOESM1_ESM.pdf]

Timescales of water accumulation in magmas and implications for short warning times of explosive eruptions.

Petrelli et al.

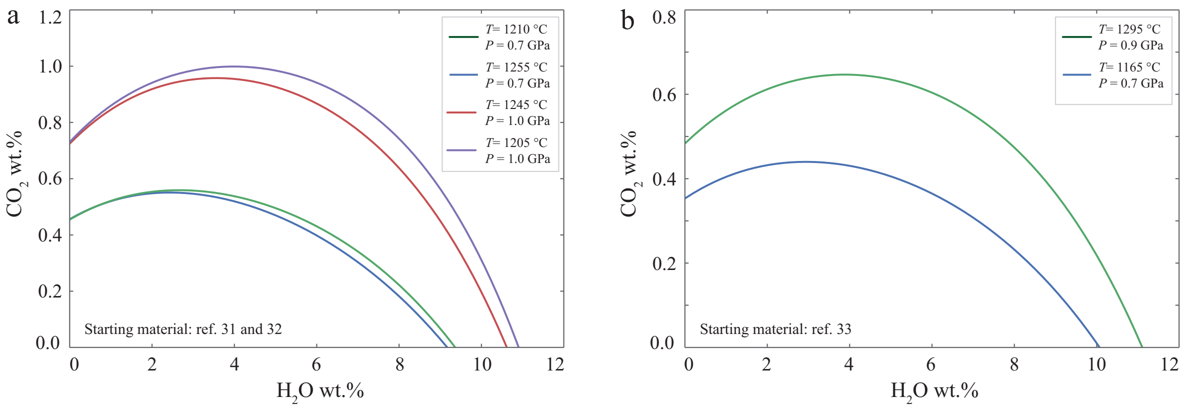

Supplementary Figure 1. **Water and carbon dioxide solubilities in the melts phase.** Solubility curves for water and carbon dioxide calculated for the (a) hig-MgO[31,32] and (b) the relatively magnesian[33] systems. All the solubility curves are calculates for the liquidus temperature of the different experimental runs reported in Supplementary Table 1.

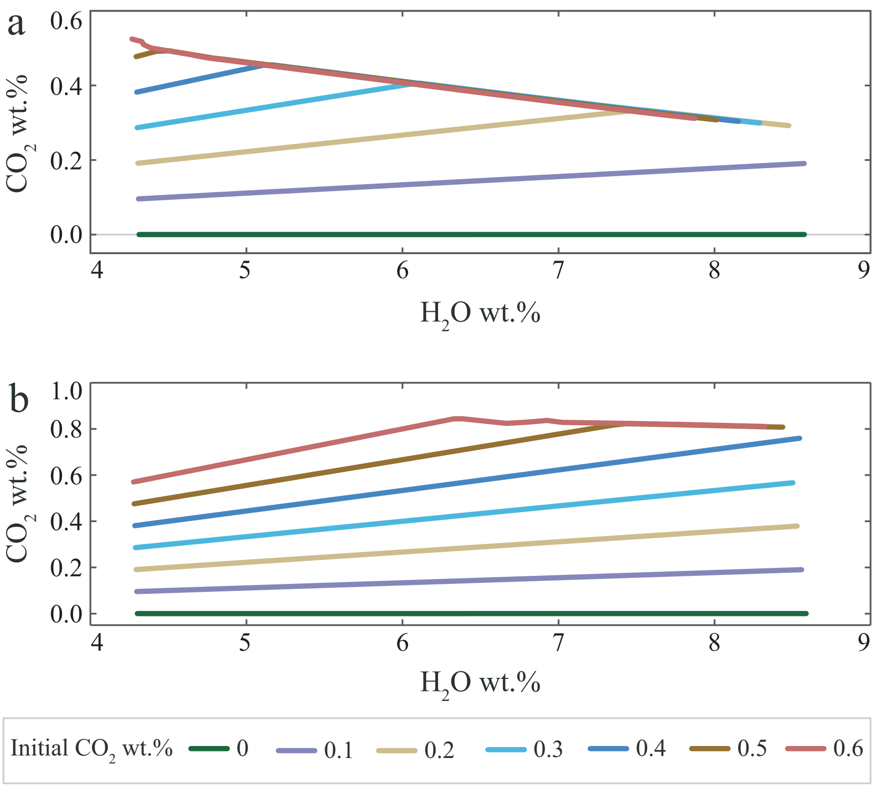

Supplementary Figure 2. **Evolution of water and carbon dioxide in the melt phase during crystallization.** Equilibrium thermodynamical models performed by rhyolite-Melts[34] displaying the evolution of H_2O and CO_2 in the residual melt during the crystallization process starting with a nominal water content of 4.5 wt.%, a) at 1.0 GPa and b) at 0.7 GPa.

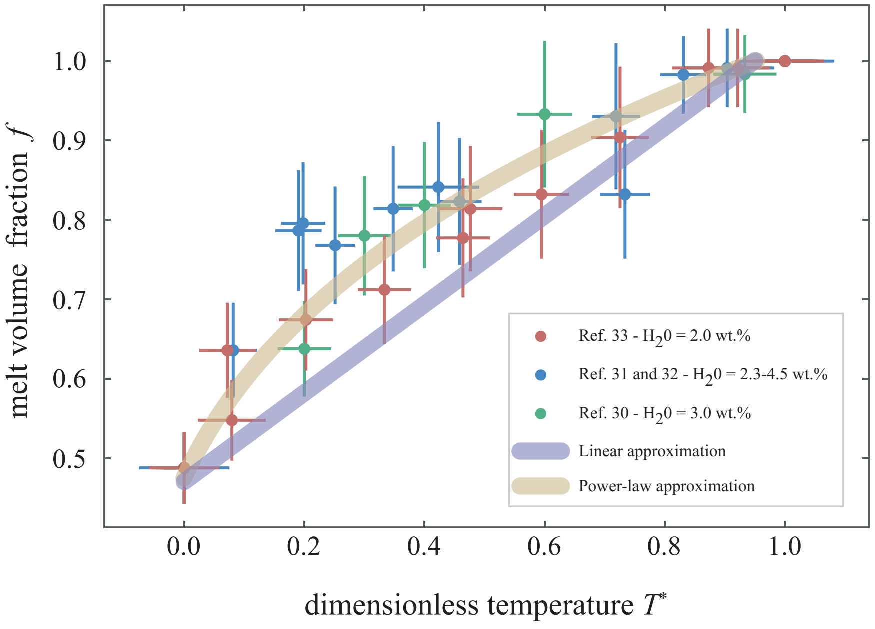

Supplementary Figure 3. **Relationship between temperature and melt volume fraction.** Relation between the volume fraction of the melt f and the dimensionless temperature for the petrological dataset utilized to constrain the parametrization of thermal numerical simulations. Error bars are reported as one standard deviation and, propagated through the equations, if required. Two main relations linking f vs. T^* have been investigated. The first is a linear approximation, $f = 0.47 + 0.558T^*$, and the second is a power-law parametrization, $f = [(T^* - T_s - 0.8)/(T_l - T_s - 0.8)]^{0.3}$, where T_s and T_l are equal to -0.886 and 0.953 respectively.

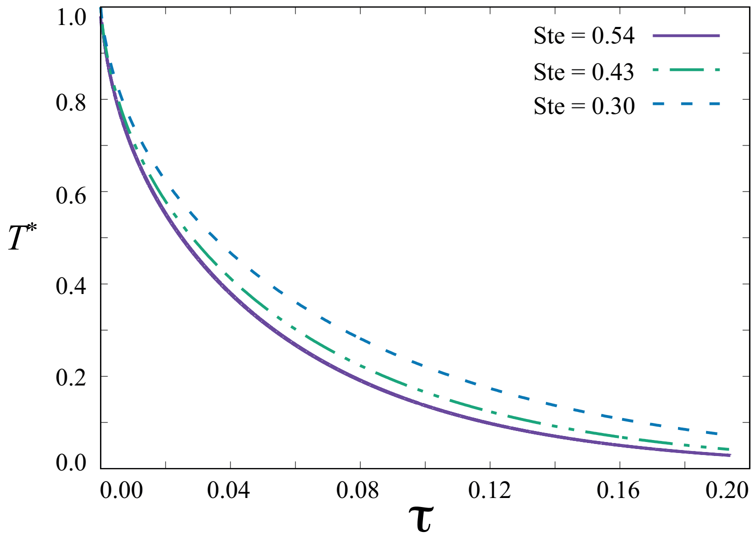

Supplementary Figure 4. **Effects of Stefan number variations on the thermal evolution of the system.** Evolution of the mean non-dimensional temperature T^* of the magma chamber with the non-dimensional time τ for three values of the Stefan number (Ste), including the maximum and minimum values (0.54 and 0.30) of the studied dataset (Tab. 1) and the typical value used for the presented simulations (0.43). Comparison is based on the static closed chamber model. Comparing, for example, the time needed to reach the value $T^* = 0.1$ the relative difference between the times found for Ste $= 0.30$ and Ste $= 0.43$ is 19% while between Ste $= 0.43$ and 0.54 is 12%.

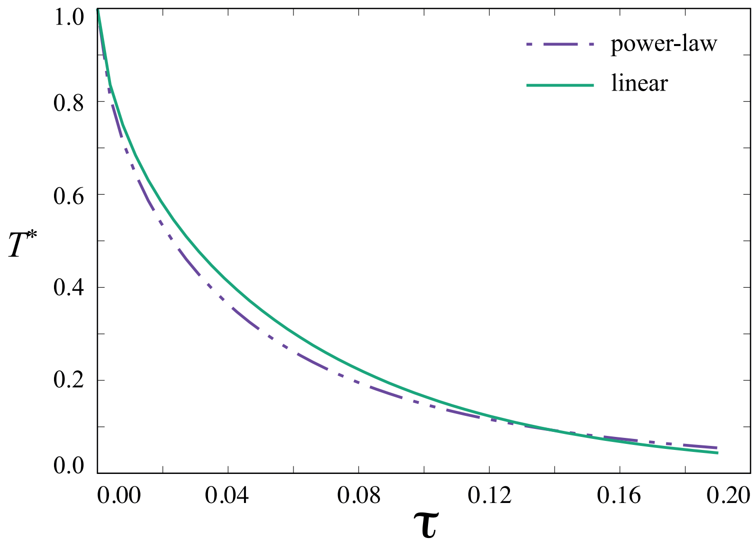

Supplementary Figure 5. **Effects of different parameterizations on the thermal evolution of the system.** Evolution of the mean non-dimensional temperature T^* of the magma chamber with the non-dimensional time τ using two f vs. T^* relations (Supplementary Fig. 4): a linear interpolation $f = 0.47 + 0.558T^*$ and a power-law interpolation $f = [(T^* - T_\mathrm{s} - 0.8)/(T_\mathrm{l} - T_\mathrm{s} - 0.8)]^{0.3}$ of the studied dataset, where T_s and T_l are equal to -0.886 and 0.953 respectively. Comparison is based on the static closed chamber model and Ste = 0.43.

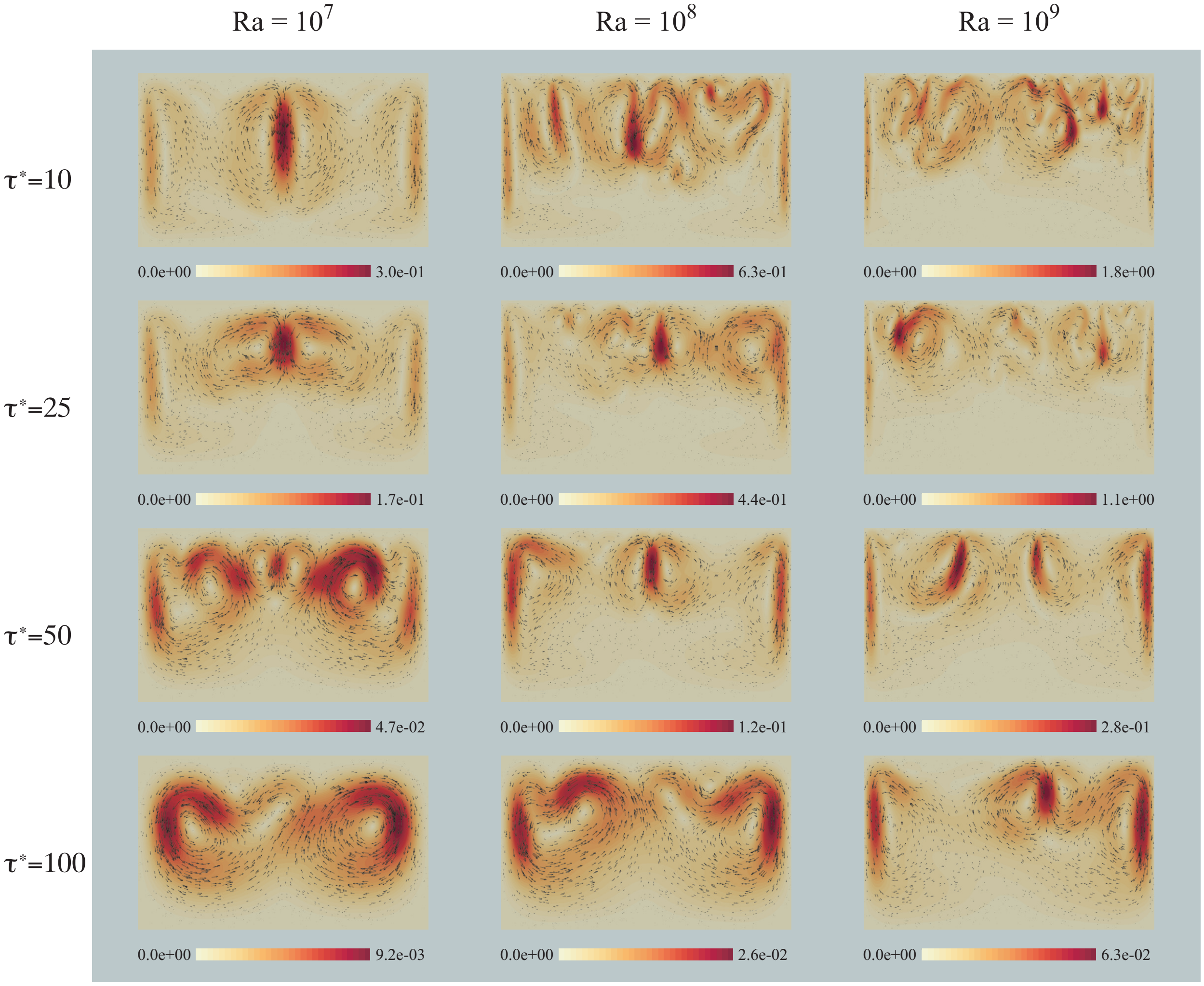

Supplementary Figure 6. **Evolution of the velocity field in the magmatic system.** Snapshots of velocity magnitude and velocity vectors for Ra $= 10^7$, 10^8 and 10^9 at different times τ^*. The parameter $\mathbf{V}$ is the dimensionless velocity as reported in equations (12), (13) and (14). Color and vector scales were changed from one figure to another because of large differences.

Supplementary Table 1. **Petrological dataset.** Petrological dataset utilized to constrain the parametrization of the numerical simulations. H_2O^i, initial water content; T_i, initial temperature (= T_r reference temperature); T_f, final temperature; $\Delta T_r = T_i - T_f = T_r - T_\infty$; ρ_r, density; η_r, dynamic viscosity; α_r, thermal diffusivity; Pr, Prandtl number; Ste, Stefan number. The thermophysical properties are considered at T_r.

Reference	Pressure [GPa]	H_2O^i [wt.%]	T_i [^{o}C]	T_f [^{o}C]	ΔT_r [^{o}C]	ρ_r [kg m^{-3}]	η_r [Pa s]	α_r [m^2 s^{-1}]	Pr	Ste
Blatter et al., 2013 [33]	0.7	2.0	1165	1040	125	2650	8.00	8.3×10^{-7}	3636	0.36
Melekhova et al., 2015 [31]	0.7	2.3	1255	1065	190	2650	1.20	8.3×10^{-7}	545	0.54
Nandedkar et al., 2014 [30]	0.7	3.0	1160	1010	150	2750	5.67	8.0×10^{-7}	2577	0.43
Melekhova et al., 2015 [31]	0.7	4.5	1210	1105	105	2540	1.60	8.7×10^{-7}	727	0.30
Blatter et al., 2013 [33]	0.9	2.0	1195	1045	150	2700	5.60	8.1×10^{-7}	2545	0.43
Melekhova et al., 2015 [31]	1.0	2.3	1245	1065	180	2700	1.20	8.1×10^{-7}	545	0.51
Melekhova et al., 2015 [31]	1.0	4.5	1205	1030	175	2590	1.70	8.5×10^{-7}	773	0.50

Supplementary Table 2. **Chemical composition of starting materials.**

Sample	RSV49	RDC156	01SB-872
SiO_2	47.44	49.92	50.29
TiO_2	0.75	0.70	1.19
$Al2O_3$	14.48	15.66	17.1
FeO	8.96	8.38	9.31
MnO	0.16	0.15	0.16
MgO	14.56	10.44	8.27
CaO	10.74	12.11	10.1
Na_2O	2.28	1.92	2.92
K_2O	0.24	0.61	0.48
P_2O_5	0.08	0.10	0,18
Cr_2O_5	0.26	nd	nd
NiO	0.04	nd	nd
Total	100.0	100.0	100.0
Reference	Melekhova et al., 2015 [31]	Nandedkar et al., 2014 [30]	Blatter et al., 2013 [33]

Supplementary Table 3. **Parametrization of the melt migration process.** Rheological parameters used to estimate melt migration velocities[70]

Property	Value	Unit
Density Contrast, $\Delta\rho$	300-500	kg m^{-3}
Fracture toughness, K_c	10^9	GPa $m^{0.5}$
Shear Modulus, G	$4.5 \cdot 10^{10}$	Pa
Poisson's ratio, ν	0.25	-
Excess Pressure, ΔP_{excess}	2-3	MPa
Bubble-free melt, ρ_m	2600	kg m^{-3}
Surrounding rock density, ρ_s	2900	kg m^{-3}
